# Flash Behaviour Does Not Help Rabbits Evade Human Predators

**DOI:** 10.1002/ece3.74609

**Published:** 2026-10-29

**Authors:** Elisabeth G. Chapman, Zenia S. W. Wong, Samuel Ellis

**Affiliations:** ^1^ Centre for Research in Animal Behaviour, Department of Psychology University of Exeter Exeter UK

**Keywords:** anti‐predator strategy, European rabbit, flash behaviour, fleeing, hidden colouration, *Oryctolagus cuniculus*

## Abstract

A number of otherwise cryptic species have areas on their body of conspicuous colour that are hidden while stationary but displayed when moving. A suggested function of this conspicuous colouration is for flash behaviour, in which an otherwise cryptic prey exposes obvious colours during escape to disrupt the predators' ability to locate the prey when it stops moving and hides the colour. Experimental studies of artificial prey with human and avian predators have found that flash behaviour can increase ‘prey’ survival, but this has not been comprehensively tested using real prey. European rabbits ( *Oryctolagus cuniculus* ) display their white tail when running but are otherwise cryptic when resting or foraging. We experimentally tested whether flash behaviour inhibits detection by presenting videos of wild rabbits fleeing to human participants and manipulated the tail colour of the rabbits. We found that rabbits with unmanipulated white tails were neither more likely nor faster to be located than rabbits with tails digitally manipulated to be cryptic. This does not support the hypothesis that flash behaviour helps rabbits evade predators.

## Graphical abstract

European rabbits display their white‐tail when running, but are otherwise cryptic when resting or foraging‐ but the function of this conspicuous colouration is unclear. This white‐tail could act as ‘flash behaviour’‐ an obvious marker shown to predators but then suddenly hidden causing the predator to lose track of the prey. We tested this using edited footage of rabbit escapes presented to human ‘predators’. We found no evidence that flash behaviour helps rabbits escape human predators.

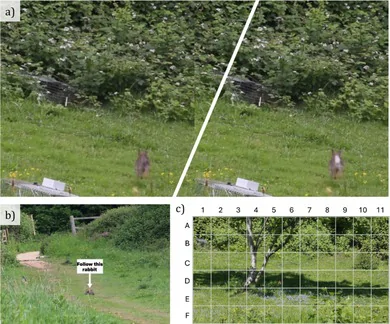

## 1 Introduction

An important way many animals avoid predation is through crypsis: having a body colour that matches the background (Ruxton et al. 2018). At the extreme, this camouflage can take the form of complex body patterns and shapes to closely match particular backgrounds (e.g., leaf mimicry in *Monocirrhus polyacanthus* fish; lichen mimicry in some *Griposia* moths; liana mimicry in *Oxybelis* tree‐snakes (Cott 1940)) or dynamically changing body colour to match the current background (e.g., rainbow fish *Melanotaenia australis* (Kelley et al. 2017), Moorish gecko *Tarentola mauritanica* (Vroonen et al. 2012), prawns *Hippolyte obliquimanus* (Duarte et al. 2018)). However, more usually, crypsis takes the form of a general matching of the colour of the background (Cott 1940). For example, mammals are typically dull‐brown or grey, which is assumed to be a form of crypsis (Caro and Mallarino 2020).

A striking feature of a number of otherwise cryptic species is that they have one or multiple areas of their body with a contrasting and often very conspicuous colour that can be revealed through behaviour (Drinkwater et al. 2022). For example, the Appennine yellow‐bellied toad *Bombina pachypus* has a camouflaged dark‐green/brown dorsal side, but when attacked can contract dorsal muscles to suddenly arch their body, exposing their bright‐yellow ventral surface to the predator (Chiocchio et al. 2024). These sudden displays of colour in response to predation threat are taxonomically widespread, and have been hypothesised to have a variety of non‐exclusive functions such as: triggering a startle reflex in the predator creating time for the prey to escape (Drinkwater et al. 2022), communicating the threat to conspecifics (e.g., Alvarez et al. 1976; Huang et al. 2025) or confusing the predator as to the preys' true location, speed or trajectory (Tan et al. 2024).

One hypothesised anti‐predator function of these contrast colour patches is as a cognitive trick on the predator: by displaying a contrasting colour while escaping, the predator focuses on this contrasting patch, the prey then hides the colour, and the predator then loses track of the location of the prey, allowing the prey to escape (Weir 1869; Walsingham 1890; Cott 1940; Marson 1945; Edmunds 1974). This hypothesised strategy, now usually known as ‘flash behaviour’, has been widely discussed but has rarely been explicitly tested. The term flash behaviour has been used in a variety of contexts, but here, following recent literature, we define it as a behaviour in which ‘an otherwise cryptic prey exhibit conspicuous colouration or noise when fleeing from potential predators […] to hinder [the] location of prey once they become stationary’ (Loeffler‐Henry et al. 2018). Potential examples of flash behaviour include the conspicuous white rump of many deer species (e.g., white‐tailed deer *Odocoileus virginianus* (Caro et al. 1995)), the red underside of female *Herennia ornatissima* spiders (Marson 1945) and the coloured tails of some Australian birds (Loeffler‐Henry et al. 2021). A series of recent studies have used human or avian predators hunting digital ‘prey’ to demonstrate that showing and then hiding a conspicuous colour increases the probability of prey survival relative to a prey that remains cryptic or conspicuous (Loeffler‐Henry et al. 2018, 2021; No et al. 2024). However, to date, no studies have investigated the efficacy of flash behaviour in enhancing the survival of real prey.

European rabbits *Oryctolagus cuniculus* are an ideal system in which to study the role of flash behaviour in helping prey escape predation. Rabbits are prey to many predators, for example, in the United Kingdom rabbits are subject to predation from species including red foxes *Vulpes vulpes* , European badgers *Meles meles* , weasels *Mustela nivalis* , birds of prey (e.g., buzzards *Buteo buteo* ), domestic cats *Felis catus* , domestic dogs *Canis lupus familiaris* and humans (Thompson and Worden 1956). Reflecting this, evading predation has been identified as a key evolutionary driver of many aspects of rabbits' behaviour (Villafuerte and Moreno 1997; Monterroso et al. 2013), morphology (Lieber and Blevins 1989; Simons 1999) and life history (Marín‐García and Llobat 2021). Flash behaviour has been hypothesised to be an anti‐predator strategy in European rabbits (Huang et al. 2025). While wild rabbits are generally cryptic, both sexes have a conspicuous white underside to their tail, which is hidden at rest but displayed when the tail is raised while running. Moreover, during 79% of escape attempts, solitary fleeing rabbits lower their tail when they stop running, hiding the white‐patch, which is consistent with flash behaviour (Huang et al. 2025). However, like other species with conspicuous white areas, it remains unknown if this behaviour is effective in helping rabbits avoid being detected, from the point of view of the potential predator.

In this study, we use an experimental approach and human‐predators to investigate whether flash behaviour can help prey avoid predation. Specifically, we compare the relative detection probability and latency of human‐predators in finding European rabbits in unedited videos (with flash effect) and videos edited to hide the white tail area (removing the flash effect).

## 2 Methods

In this study, we first filmed escape behaviour in wild rabbits (*Filming*), then edited the footage to include or not include flash behaviour (*Video selection and editing*), and then ran an online experiment using human participants to ‘hunt’ rabbits in the edited and unedited videos (*Online experiment*).

### 2.1 Filming

We obtained videos of rabbits by filming a population of wild European rabbits living on the University of Exeter Streatham campus in Exeter, UK. We filmed rabbits of all age classes, some of which were marked for identification as part of ongoing research. The university campus contains extensive green space and natural habitats, is on the edge of the city with some boundaries directly adjacent to open farmland and is subject to natural predation pressures. We have noted the presence of red foxes, European badgers, buzzards, domestic cats and dogs (EGC pers. obs.) in the study area, all of which are known to hunt rabbits (Thompson and Worden 1956).

Filming was carried out by a single observer (ZSW) in two time‐blocks: midday (10:00–14:00) and evening (17:00–20:00) for 2 weeks in May 2025. These times were selected to ensure full daylight for high‐quality footage. Rabbits in this population are not strictly crepuscular and are commonly seen foraging during daylight hours (EGC, SE pers. obs.). Filming was carried out using a Canon EOS1200D camera mounted on a tripod. The observer aimed to film from approximately 20 m distance from the rabbit, but this was not always possible due to the structure of the environment and rabbit behaviour. Previous work in this population has demonstrated that rabbits' vigilance rate (as a measure of perceived risk) is not influenced by the distance they are from observers greater than 9 m away (Chapman et al. *in prep*).

The observer directed the camera towards a focal rabbit and began filming when a natural threat (e.g., foxes, dogs, or humans) was present in the area, causing rabbits to flee to cover. Overall, 290 videos of rabbits fleeing were captured, ranging from 5 s to 3 min in duration.

### 2.2 Video Selection and Editing

Of the 290 videos captured, 13 were selected for the experiment that fitted one of the following criteria: (1) the focal rabbit ran completely into cover (usually dense bramble bushes *Rubus fruticosus* ) where it was completely concealed (*n* = 3); (2) the focal rabbit ran into cover but was still partly or completely visible (*n* = 4); or (3) the focal rabbit ran and stopped in the open (*n* = 6). These were trimmed to 3–5 s in length to capture only the fleeing part of each video. Videos with multiple rabbits running or containing dogs were removed, but videos containing other rabbits foraging or resting were included. The selected videos included rabbits fleeing on a grass background or on a gravel path with grass and bramble bushes on either side. Running angles and sizes of the rabbits also varied between videos, but in all selected videos, the white underside of the tail was visible while the focal rabbit ran.

Selected videos were edited in Filmora v14.7.11 (Wondershare 2025). We cropped videos to 1934 × 1088 pixels, removed the sound and duplicated each video to obtain an edited and an original version of each video. For the edited videos, using the mask function in Filmora, we covered the white patch on the underside of the tail with a shade of brown matching the body colour of the focal rabbit (Figure 1a). The video editing resulted in 26 videos for use in our online experiment: 12 experimental videos with a cryptic‐tail (edited) and white‐tail (unedited) versions (*n* = 24), and one control video with an original (unmanipulated) and a manipulated version (*n* = 2) (see *Manipulation Control*).

**FIGURE 1:**
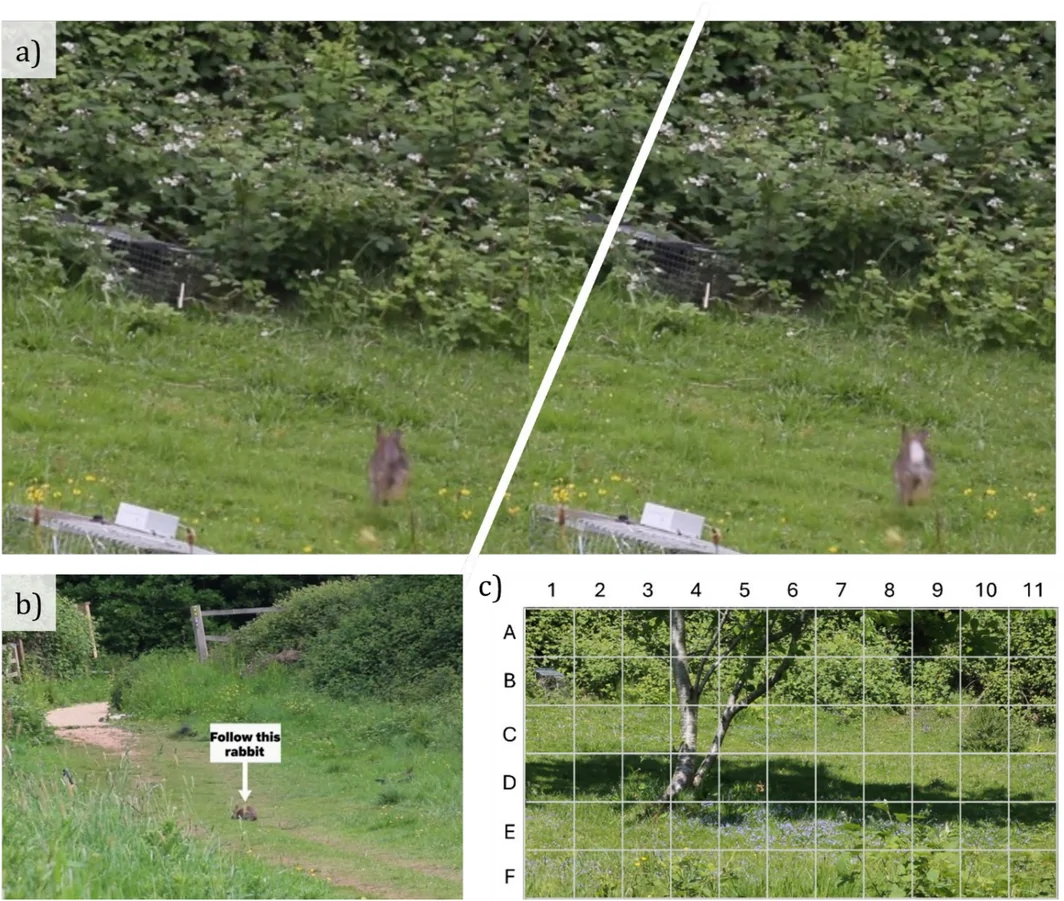
Summary of the experimental setup. (a) Participants were presented with videos of rabbits that were either edited so a brown patch covers their white tail (cryptic‐tail; left), or an unedited video with their natural white‐tail (right). (b) First frame of the video containing an arrow indicating the ‘focal rabbit’ for participants to follow. Participants were then presented with a video, either with an original white tail or edited to a cryptic brown tail. (c) Following the video, and a black screen for 1 s, the last frame of the video was presented (regardless of whether or not the focal rabbit was in view), with a grid overlaid, participants were asked to select one grid cell that they thought contained the focal rabbit. Individuals were presented with twelve videos following this format.

### 2.3 Online Experiment

Human participants (*n* = 183) were recruited using Prolific (www.prolific.com) between July and August 2025. Prolific is a widely used online platform for recruiting participants for research studies. To take part in our study participants were required to be over 18 and fluent in English and to be taking part from a desktop computer (i.e., not a phone or tablet), no other exclusion criteria were applied. Participants chose to take part in the study after reading a title (‘Tracking animal movement’) and short general description of the study. The description gave the general aims of the study as ‘how accurately predators visually track the movement of prey species’ but did not give further details (Figure S1). Each participant was paid £1.80 for participating in the study (equivalent to ~£10.80 per hour), which aligns with Prolific's fair payment principles. Data were collected anonymously and all participants gave their consent for their responses to be used for this study. Participants were given a short demographic survey after the experiment (Table S1).

We based our experiment on an object‐tracking game previously used in studies of flash behaviour (Loeffler‐Henry et al. 2018), which we adapted for use with a natural prey. We used Gorilla Experiment Builder software (Anwyl‐Irvine et al. 2020) to create the online experiment, the aims and hypotheses of which were not disclosed to participants. Each participant was presented with six cryptic‐tail (edited) and six white‐tail (unedited) videos, plus the manipulation control (see below). No participant received both the edited and original versions of the same video. Participants were randomly allocated to one of ten ‘order sets’. We manually created these order sets because Gorilla Experiment Builder did not allow a unique order to be generated for each participant. Each order set contained six cryptic and six white‐tailed videos and manipulation control video in a random order.

Prior to the trials, instructions were provided to participants, with an example video to ensure understanding. An experimental trial consisted of participants watching the video and following the target rabbit. Before each trial, a still image of the first frame of the video with the target rabbit identified with an arrow was shown to the participants for five milliseconds, with the video automatically playing immediately after (Figure 1b). At the end of the video, participants were presented with a black screen for 1 s, followed immediately by the last frame of the video (regardless of whether the rabbit was visible in this frame). The image was overlaid with a labelled grid (6 × 11) (Figure 1c). Participants were then asked to indicate the grid cell where the rabbit was last seen by selecting the correct row and column from two dropdown lists as their response. Participants' response time was recorded, timed from when the still image was loaded until they selected their answer from the dropdown lists and submitted their answer. In instances where the rabbit occupied two grid cells in the final image, both cells were treated as correct. In instances where the rabbit ran out of sight, participants were asked to identify the cell in which they last saw the rabbit. We asked participants not to use the computer mouse or other objects to track the rabbit and provided a ‘do not know’ option to select if participants could not find the target rabbit. In instances when participants selected ‘do not know’, these were treated as incorrect. Participants were aware that their responses were being timed.

### 2.4 Analysis

Data were analysed in R version 4.5.1 (R Development Core Team 2021). For each question, Bayesian mixed models implemented in ‘brms’ (Bürkner 2017) were used to interpret the relationship between the video type and the response variable. Unless stated otherwise, all models included random intercepts for participant ID and video ID to account for the fact that participants were presented with multiple videos, and each video was presented to multiple participants, and were implemented with weakly informative priors. We tested whether participants got better with attempts by regressing the video presentation order against detection probability and log detection latency with the error structure described above. There was no effect of this (see results), and therefore, order was not included in subsequent analyses.

We then used four metrics to measure the efficacy of flash behaviour in helping rabbits evade predators: (1) detection probability; (2) detection latency; (3) giving‐up probability; (4) giving‐up latency. Detection probability was defined as the probability that a participant successfully located the target rabbit in the video. A successful location was scored as correct if the participant correctly identified the grid cell(s) containing the target rabbit. Detection probability was modelled as a Bernoulli process with tail‐colour (cryptic‐tail vs. white‐tail) as the fixed effect and the random effect structure described above. For likelihood of detection analyses, the data for control videos were excluded from the analysis.

Detection latency was measured as the time taken for a participant to successfully identify the target rabbit's location. These data were log‐transformed for these analyses. Log detection latency was modelled as a Gaussian distribution with tail‐colour as the fixed effect and the random effect structure described above. For this model, instances where the participant failed to locate the focal rabbit were excluded from the analysis. To validate that latency was a useful metric for the difficulty of locating the rabbits in the environment we tested the relationship between latency and the probability that a response is correct. If latency was a useful measure of predator behaviour, we expected that participants would take longer to respond correctly than incorrectly. This was also modelled as a Gaussian distribution with detection response (correct or incorrect) as the fixed effect and the error structure described above predicting the log response time.

Giving‐up probability was modelled as a Bernoulli process with the model structure described above. Participants were considered to have given up if they selected ‘do not know’ for one or both of their dropdown responses. Giving‐up latency was defined as the time taken for the participants to select ‘do not know’ from the drop‐down options. As for detection latency, we ran a model with a Gaussian distribution, with log‐transformed response time as the response variable, tail‐colour as the fixed effect and random effect structure as outlined above. This analysis only included instances where participants selected ‘do not know’ for one or both grid axes (*n* = 41).

We also tested the effect of video ID on detection probability and latency to determine if some videos were more or less difficult than others. We ran a model with a Bernoulli distribution with detection probability as the response variable and video ID as the predictor. Similarly, we ran a model with a Gaussian distribution to determine whether video ID influenced log‐transformed response time. Tail colour and participant ID were included as random intercepts for these models. We then reran the detection probability and detection latency analyses the same way as described above, but including only videos identified as more difficult.

Finally, we tested the effect of video type (whether or not the rabbit remained in view at the end of the video) on detection probability and latency. We ran a model with a Bernoulli distribution with detection probability as the response variable and whether the rabbit remained in view as a binary predictor variable. We also ran a model with a Gaussian distribution with log‐transformed response time (if correct) as the response variable and whether the rabbit remained in view as the predictor variable. Participant ID and video ID were included as random intercepts in these models. We then reran the effect of tail colour on detection probability and detection latency as described above, but including only videos where the rabbit remained visible in the last frame.

The models we used are a Bayesian implementation of regression models. These use a Hamiltonian Monte Carlo algorithm to explore the parameter space and instead of producing a single point estimate generate a range of plausible values for each parameter given the data. The result is a distribution of potential estimates (the posterior distribution) from which we extract estimates in the form of mean estimates or draws (individual estimates) while maintaining uncertainty. For all models, we report the coefficient of interest as the mean of the posterior distribution (*β*) with the 95% credible intervals (CI). We also report the proportion of draws from the posterior that show positive or negative relationships by dividing the number of draws that are positive or negative by the total number of draws. This is the probability that the coefficient is above or below zero and therefore the probability that the relationship is positive or negative (reported as *P*(*β* > 0) or *P*(*β* < 0)). For the tail colour term, the parameter is the difference in probability/latency of detection between videos with white and cryptic tail colours.

### 2.5 Manipulation Control

We included a manipulation control to ensure that the process of editing the videos did not make the rabbits more or less conspicuous. In the manipulation control the natural tail colour of the rabbit (white) was replaced with a different shade of white rather than made cryptic. We used the mask function in Filmora to create the manipulation control (as described above); once outlined, the colour of the tail was manipulated by changing ‘brightness’, ‘blur strength’ and ‘whiteness’ settings of the enclosed area to generate a new but biologically realistic shade of white based on the original colour. Each participant was presented with either the manipulated or original control video.

We fitted models to determine whether editing the videos influenced detection probability (Bernoulli distribution) or log detection latency (Gaussian distribution). In these models, the fixed effect was whether the participants were presented with the manipulated or unmanipulated version of the experimental control video, and the response was detection probability or logged response time. For this, participant ID and video ID were not included as random effects, as each participant was only shown one of the two control videos, and there was only one video used.

### 2.6 Ethical Note

The study was reviewed and approved by the University of Exeter Psychology Ethics Committee (reference: 9349855).

## 3 Results

183 participants took part in the online experiment, each presented with 12 experimental and 1 control video (*n* = 2196 experimental trials). Across all participants for the experimental videos, there were 1851 correct responses and 345 incorrect responses. Participant ID explained 19.6% of variance in detection probability and 50.8% of the variance in detection latency. There was no effect of video presentation order on detection probability (*β* = −0.09, 95% CI [−0.41, 0.24], *P*(*β* < 0) = 0.73), or detection latency (*β* = 0.00, 95% CI [−0.01, 0.02], *P*(*β* > 0) = 0.57). The experimental videos were of at least six individual rabbits. There was no relationship between rabbit ID and detection probability (all 95% CI overlap between 0.57–1), or detection latency (all 96% CI overlap between 8.74 and 9.34).

### 3.1 What Is the Effect of Tail Colour on Detection Probability?

There was no difference in the likelihood of being located between rabbits with cryptic‐tails and rabbits with white‐tails. Participant success was not predicted by the tail‐colour in the videos (*β* = −0.10, 95% CI [−0.41, 0.22], *P*(*β* < 0) = 0.72; Figure 2a).

**FIGURE 2:**
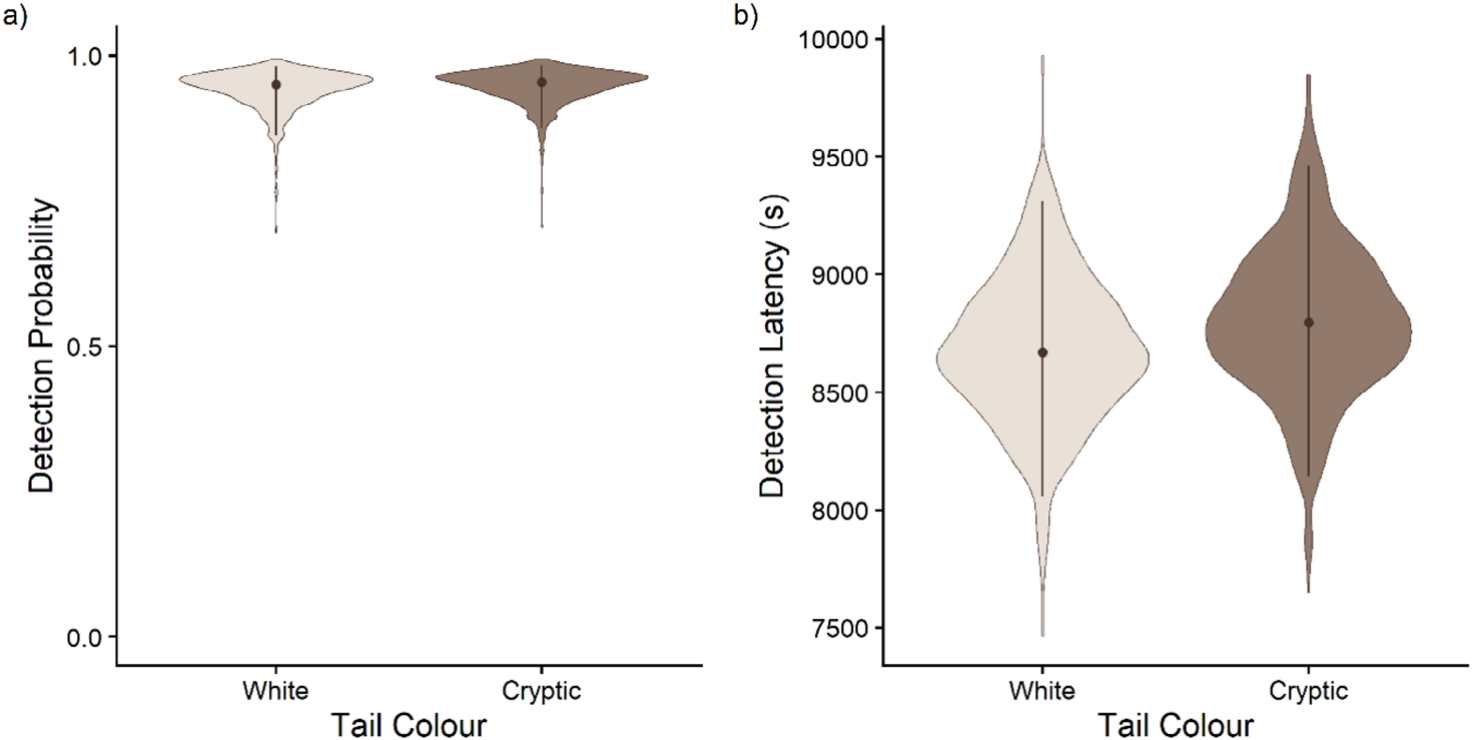
Effect of tail colour (unedited white‐tail, edited cryptic‐tail) on (a) probability of detecting the rabbit (detection probability; n white condition = 1081, n cryptic condition = 1115), and (b) the time to record a correct response (detection latency; n white condition = 907, n cryptic condition = 944). Violins show the distribution of 500 random draws from the posterior distribution. The brown dots show the median estimate, with 95% credible intervals.

### 3.2 What Is the Effect of Tail Colour on Detection Latency?

Similarly, response time for correct responses was not predicted by the tail‐colour (*β* = −0.01, 95% CI [−0.04, 0.01], *P*(*β* < 0) = 0.88; Figure 2b). However, participants did take longer to respond when they were incorrect than when they were correct, regardless of tail‐colour (*β* = −0.16, 95% CI [−0.20, −0.11], *P*(*β* < 0) = 1).

### 3.3 What Is the Effect of Tail Colour on Giving‐Up Probability?

Videos of rabbits with brown tails were no more or less likely to elicit a giving‐up response than videos of rabbits with white flashing tails (*β* = −0.37, 95% CI [−1.15, 0.39], *P*(*β* < 0) = 0.83). Similarly, tail colour was not associated with a difference in giving‐up latency (*β* = 0.15, 95% CI [−0.10, 0.38], *P*(*β* > 0) = 0.89).

### 3.4 Do Detection Probability and Detection Latency Depend on Video Difficulty and Type?

Video ID influenced the detection probability of participants. Three of the twelve videos had credible intervals outside of the range of the other nine, indicating they were more likely to elicit an incorrect response (Video ID 1481: 95% CI [0.46, 0.84], Video ID 1493: 95% CI [0.44, 0.83] and Video ID 1702: 95% CI [0.12, 0.46], all other videos: 95% CI between 0.85 and 1). Re‐running the analyses on only these ‘difficult’ videos, there remained no effect of tail‐colour on detection probability (*β* = −0.03, 95% CI [−0.42, 0.36], *P*(*β* < 0) = 0.56) or detection latency (*β* = 0.00, 95% CI [−0.05, 0.05], *P*(*β* < 0) = 0.50). There was no difference in detection latency in any of the videos (all videos had overlapping credible intervals: 95% CI between 8.26 and 9.71).

Participants were more likely to correctly locate the focal rabbit when it remained completely or partially in view at the end of the video (*β* = 2.43, 95% CI [0.72, 3.77], *P*(*β* > 0) > 0.99). However, there was no difference in detection latency based on whether the rabbit was in view at the end of the video (*β* = −0.07, 95% CI [−0.21, 0.06], *P*(*β* < 0) = 0.86). When we restricted the data to ask how participants performed when the rabbit remained in view, there remained no effect of tail colour on detection probability (*β* = −0.13, 95% CI [−0.67, 0.40], *P*(*β* < 0) = 0.67) or detection latency (*β* = −0.01, 95% CI [−0.04, 0.01], *P*(*β* < 0) = 0.82).

### 3.5 Manipulation Control

Neither detection probability nor latency was dependent on the editing process: participants were no more or less likely to respond correctly to edited or unedited control videos (*β* = −0.32, 95% CI [−1.30, 0.57], *P*(*β* < 0) = 0.77). Participants did not differ in their response time between edited and unedited control videos (*β* = −0.02, 95% CI [−0.14, 0.09], *P*(*β* > 0) = 0.62).

## 4 Discussion

We find no evidence that flash behaviour increases the chance that rabbits will remain undetected by human predators. Specifically, we found no difference in the relative likelihood of participants successfully locating prey (detection probability) or time taken to locate the prey (detection latency) between videos showing rabbits with cryptic or white tails. Participants also did not show a higher giving‐up probability or take longer to give up based on whether the videos presented showed a rabbit with a white or cryptic tail.

Previous research in European rabbits has found that the behaviour of fleeing rabbits is consistent with multiple functions including flash behaviour (Huang et al. 2025). In a detailed study using experimentally‐induced escapes in wild rabbits, Huang et al. (2025) found that 71% of escaping individuals hid their tail once they stopped running, and that rabbits further away from their ‘predator’ were more likely to hide their tail post‐flight than rabbits closer to their predator (Huang et al. 2025). Both of these results are consistent with flash behaviour being used during escape. However, despite these consistencies, the authors found more support for other functions of rabbit tail flashing, principally as a warning to conspecifics (particularly kits and juveniles) and potentially as a quality advertisement signal to the predator deterring pursuit (Huang et al. 2025). Our results agree with the conclusions of this study that flash behaviour does not play a significant role in the anti‐predator adaptations of European rabbits.

Our results also add important context to comparative studies, which have found mixed evidence that the conspicuous colouration in animals has evolved to facilitate flash behaviour. For example, across lagomorphs, white tails are not more common in species with more cryptic body colouration, which suggests that white tails did not evolve for flash behaviour in this taxon (Huang et al. 2025). In Artiodactyla, on the other hand, conspicuous colouration that can be facultatively displayed is more common in less social species, which is consistent with a non‐collective antipredator role such as flash behaviour (Caro et al. 2020).

To our knowledge, this is the first study to test the adaptive significance of flash behaviour using real prey from the point of view of a potential predator. Our results differ from previous studies using simulated prey, which have found that flash behaviour does increase the survival of prey (Loeffler‐Henry et al. 2018, 2021; No et al. 2024). There may be several non‐exclusive explanations for the differences between our results and these previous results. First, it could be that the rabbits were easier for the human predators to track than the prey presented in the simulation experiments. In the simulation experiments, prey are small squares against a background of grass or sand (Loeffler‐Henry et al. 2018, 2021). Humans are predators of rabbits (Calvete et al. 2005; Jones 2006), and even in a modern context, many participants may have some experience of observing rabbits or similar species in the wild or in a domestic setting (e.g., 32% of participants had a pet rabbit at the time of the experiment, or in the past; Table S1). This evolutionary and individual experience could mean that humans are better at assessing the direction and trajectory of rabbits than the simulated prey, meaning the benefits of flash behaviour were negligible; indeed, the success rate of participants at finding the rabbits in our experiment was very high (mean = 84.3% correct). Secondly, the studies with simulated prey show that the efficacy of flash behaviour is affected by context. For example, flash behaviour is more effective if escape is initiated when the predator is far away (Loeffler‐Henry et al. 2021) and when the prey are larger (No et al. 2024). In our experiments, all videos were filmed from between approximately 10 and 20 m, so there was limited variation in the distance from and size of rabbits in our videos. Further experiments, varying the distance the footage is taken from, would give insight into whether flash behaviour is more effective at longer distances. Thirdly, although in our experiments the prey are ‘real’, the predation scenario is still *in silico*. Real predation events could differ in ways that may affect the conclusions of our experiment. For example, humans vision differs in several ways, particularly chromacy and acuity, from other important rabbit predators (e.g., Canidae (Mowat and Peichl 2022); Felidae (Corsi et al. 2022); Aves (Jones et al. 2007)). Future work could explore how the efficacy of rabbit flash behaviour differs from the perspective of different visual systems. The human predators in our experiment were also limited to visual cues, whereas natural predation events are likely to rely on multimodal sensory information (e.g., Roberts et al. 2007; Rhebergen et al. 2015). Similarly, whereas in our experiment, prey are moving relative to a stationary point (the camera), in real predation events, both predator and prey will be moving, which may change the sensory and cognitive processing of the flash behaviour.

Flash behaviour is an intuitive idea to explain the mystery of why otherwise cryptic species make themselves conspicuous during predator escape. However, our results add to the generally mixed evidence for flash behaviour as an anti‐predator strategy (Loeffler‐Henry et al. 2018, 2021; Caro et al. 2020; No et al. 2024; Huang et al. 2025). There are several ecological and evolutionary complexities that may help explain this mixed evidence. Ecologically, the mechanism of flash behaviour requires a pursuit predator. However, most prey species will be subject to multiple predation strategies: European rabbits in the UK, for example, will be subject to ambush predation (Moseby and McGregor 2022), being hunted underground (Brown and Lasiewski 1972), as well as pursuit predation (Broseghini et al. 2024). The same trait may also have different functions at different phases of a predation sequence. Evidence suggests that rabbit white‐tails may act as an alarm signal to conspecifics before escape and a quality advertisement during prey pursuit (Huang et al. 2025). Anti‐predator strategies evolve in response to multiple threats and have multiple functions, and to understand the evolution of one strategy—such as flash behaviour—may require a holistic view to understand how it coevolves or constrains other strategies. Future work in other systems, species and contexts using real prey and/or real predators to investigate the efficacy of flash behaviour will give more insight into this unusual hypothesis.

## Author Contributions

**Elisabeth G. Chapman:** conceptualization (equal), data curation (equal), formal analysis (equal), investigation (supporting), methodology (supporting), supervision (supporting), visualisation (lead), writing – original draft (equal), writing – review and editing (equal). **Zenia S. W. Wong:** conceptualization (equal), data curation (equal), formal analysis (equal), investigation (lead), methodology (equal), visualisation (supporting), writing – original draft (equal), writing – review and editing (equal). **Samuel Ellis:** conceptualization (equal), data curation (supporting), formal analysis (supporting), methodology (supporting), project administration (lead), supervision (lead), visualisation (supporting), writing – original draft (equal), writing – review and editing (equal).

## Funding

E.G.C. was supported by a NERC GW4+ DTP studentship (NE/S007504/1: Project reference: 2859385).

## Conflicts of Interest

The authors declare no conflicts of interest.

## Supporting information


**Table S1:** Demographic summary of study participants. Note, responding to any or all of the demographic questions was optional for participant so numbers for each question do not necessarily sum to the total sample.
**Figure S1:** Landing Page for the study on prolific platform. This is the information received by participants before they chose to take part in the study.

## Data Availability

All data and code are available here: https://osf.io/ntbd4/overview?view_only=70ab1e16ed634e33b9b967a81dc313a7.
