# Supplementary material for: Flash Behaviour Does Not Help Rabbits Evade Human Predators
Source: Ecol Evol. 2026 Oct 29;16(11):e74609. doi: 10.1002/ece3.74609 (PMC13719797; doi:10.1002/ece3.74609)
Supplement: Supplementary file 1 [file ECE3-16-e74609-s001.docx]

Table S1. Demographic summary of study participants. Note, responding to any or all of the demographic questions was optional for participant so numbers for each question do not necessarily sum to the total sample.

| Question | Response | N Respondents |
| --- | --- | --- |
| How do you describe your gender? | Female | 81 |
|  | Male | 99 |
|  | Non-binary | 3 |
| Which age group do you belong to? | 18-24 | 25 |
|  | 25-34 | 65 |
|  | 35-44 | 48 |
|  | 45-54 | 22 |
|  | 55-64 | 17 |
|  | 65 or older | 6 |
| Have you ever had a pet rabbit (currently or in the past)? | No, I have never had a pet rabbit | 123 |
|  | Yes, I currently have a pet rabbit | 10 |
|  | Yes, I have had a pet rabbit in the past | 49 |
|  | No response | 1 |
| Do you have any form of colour blindness (colour vision deficiency)? | I am not sure | 4 |
|  | No, I have normal colour vision | 177 |
|  | Yes, I have a colour vision deficiency | 2 |

Figure S1. Landing Page for the study on prolific platform. This is the information received by participants before they chose to take part in the study.
